# Acidity Is an Excellent Marker of Infection in Hip and Knee Arthroplasty

**DOI:** 10.3390/jcm13030688

**Published:** 2024-01-25

**Authors:** Tobiáš Judl, Stanislav Popelka, Elena Tomšík, Martin Hrubý, Matěj Daniel, Jaroslav Fojt, Pavel Melicherčík, Ivan Landor, David Jahoda

**Affiliations:** 1 Department of Orthopaedics, 1st Faculty of Medicine, Charles University in Prague and University Hospital in Motol, V Úval 84, 150 06 Prague 5, Czech Republic; stan2005@volny.cz (S.P.); david.jahoda@post.cz (D.J.); 2 Institute of Macromolecular Chemistry CAS, Heyrovsého nám. 2, 162 00 Prague 6, Czech Republicmhruby@centrum.cz (M.H.); 3 Faculty of Mechanical Engineering, Czech Technical University, Technická 4, 166 07 Prague 6, Czech Republic; matej.daniel@fs.cvut.cz; 4 Department of Metals and Corrosion Engineering, University of Chemistry and Technology, Technická 5, 166 28 Prague 6, Czech Republic; jaroslav.fojt@vscht.cz

**Keywords:** pH, infection detection, synovial fluid, prosthetic joint infection (PJI)

## Abstract

Background: The diagnosis of joint replacement infection is a difficult clinical challenge that often occurs when the implant cannot be salvaged. We hypothesize that the pH value of synovial fluid could be an important indicator of the inflammatory status of the joint. However, in the literature, there is a lack of data on the pH changes in hip and knee joint replacements and their relation to infection and implant failure. In this study, we aimed to measure the pH levels of synovial fluid in patients with hip and knee joint replacements. We also investigated the potential of pH measurement as a diagnostic tool for joint replacement infection. In this study, we recorded the pH values to be 7.55 and 7.46 in patients where *Pseudomonas aeruginosa* was identified as the cause of the prosthetic joint infection. We attribute this to the different environments created by this specific bacterium. In other cases where the pH was higher, chronic mitigated infections were diagnosed, caused by strains of *Staphylococcus aureus*, *Streptococcus agalactiase*, and coagulase negative *staphylococcus*. Materials and methods: In our cohort of 155 patients with implanted hip (THA; n = 85) or knee (TKA; n = 70) joint replacements, we conducted a prospective study with a pH measurement. Out of the whole cohort, 44 patients had confirmed joint replacement infection (28.4%) (44/155). In 111 patients, infection was ruled out (71.6%) (111/155). Joint replacement infection was classified according to the criteria of the Musculoskeletal Infection Society (MSIS) from 2018. Based on the measured values, we determined the cut-off level for the probability of ongoing inflammation. We also determined the sensitivity and specificity of the measurement. Results: The group of patients with infection (n = 44) had a significantly lower synovial fluid pH (pH = 6.98 ± 0.48) than the group of patients with no infection (n = 111, pH = 7.82 ± 0.29, *p* < 0.001). The corresponding median pH values were 7.08 for the patients with infection and 7.83 for the patients with no infection. When we determined the cut-off level of pH 7.4, the sensitivity level of infected replacements was 88.6%, and the specificity level of the measurement was 95.5%. The predictive value of a positive test was 88.6%, and the predictive value of a negative test was 95.5%. Conclusions: Our results confirm that it is appropriate to include a pH measurement in the diagnostic spectrum of hip and knee replacements. This diagnostic approach has the potential to provide continuous in vivo feedback, facilitated by specialized biosensors. The advantage of this method is the future incorporation of a pH-detecting sensor into intelligent knee and hip replacements that will assess pH levels over time. By integrating these biosensors into intelligent implants, the early detection of joint replacement infections could be achieved, enhancing proactive intervention strategies.

## 1. Introduction

Hip and knee replacements are implanted in millions of people worldwide every year. Knee and especially hip replacement surgeries are considered among the most successful surgeries. However, despite the preoperative precautions (indications for surgery, patient type, search for current infection foci, and the preparation of the surgical field), perioperative precautions (operating room, antibiotic prophylaxis, and operator), and postoperative precautions (wound care and the prevention of hematogenous infection related to high-risk diseases or surgeries), infection in the joint replacement area occurs in 0.5–2% of patients with hip replacement and in up to 5% of patients with knee replacement [1,2,3,4] Infection in the joint replacement area is always a major problem for the patient and the orthopedic surgeon. The detection of a replacement infection is often delayed until inflammation has developed because of the minimal initial discomfort to the patient and the difficulty and complexity in detecting the infection. If bacteria are present in the joint replacement area, a bacterial biofilm will begin to form on the implant surface within a few hours, so it is important to make a diagnosis as soon as possible. In the case of an early diagnosis within 1–2 weeks from the onset of symptoms, it is possible to proceed to antibiotic treatment and debridement of the replacement area with replacement of the articulating surfaces of the implant [5]. The earlier the treatment is started, the significantly higher the chance of success, as biofilm maturation during the progress of infection makes bacteria elimination progressively harder over the course of time. In the case of a late diagnosis and a duration of infection longer than 3–4 weeks, due to the presence of a mature bacterial biofilm, it is necessary to proceed to the extraction of the implant [6,7,8]. This solution often leads to a loss of bone substrate, the need for revision implants and, in the case of an unknown bacterial agent, two-stage reimplantation using a cement spacer with antibiotics. The mortality rate is also increased in elderly patients with joint replacement infection [9].

The current diagnosis of postoperative infections is based on a combination of clinical findings based on non-specific symptoms, laboratory results from peripheral blood, microbiological samples (culture and PCR—polymerase chain reaction), histological examinations of tissue, perioperative follow-ups, and imaging techniques (such as X-ray, magnetic resonance imaging, and computed tomography) [5,10]. These investigations mainly include systemic markers of inflammation that do not localize the source of inflammation and have a poor sensitivity/specificity for implant-associated infection. Infection markers may be more sensitive and specific in synovial fluid than in serum because they are localized to the site of infection [11]. The current standard of care for the diagnosis of joint replacement infection involves synovial fluid examination, in which a syringe puncture of the joint is performed, and the white blood cell count are analyzed and differential, crystal, Gram staining and culture are conducted [12,13]. In addition, synovial fluid contains infectious biomarkers, such as glucose [14], a low pH, high lactate concentrations [15], C-reactive protein [16,17], interleukins [18], interferon-γ [10], α-defensins [16,19,20], and cathelicidins [21], which may be useful for the diagnosis of infection.

As early as 1937, Menkin described a decrease in the pH of the exudate during an ongoing infection to a value of 6.5 [22]. If sufficient synovial fluid is obtained from the area of arthroplasty, this examination can easily be performed. This method also has the advantage of obtaining a result almost instantaneously, which, together with other examinations and clinical findings, allows for an early intervention in the infection of the joint replacement.

Our goal is to obtain a fast yet clinically relevant method to confirm the infection of joint replacements as soon as possible from the onset of inflammation. In cooperation with the Institute of Macromolecular Chemistry, CAS, the University of Chemistry and Technology, Prague, and the Faculty of Mechanical Engineering, Czech Technical University in Prague, we are developing an implantable co-sensor, the main function of which will be pH detection. Therefore, the aim of this study was to confirm that the inflammation of a joint replacement is accompanied by measurable changes in the acidity of synovial fluid.

## 2. Materials and Methods

Materials: 3% hydrogen peroxide and KCl were from Sigma Aldrich, Czech Republic; Hamilton Duracal Buffers (4.01, 7, and 10.01) were from Lach-ner, Czech Republic; Columbia Agar with Sheep Blood (7%), MacConkey Agar, Chocolate Agar with Vitox (cultivated in an environment with 5% CO_2_), and Thioglycolate Broth were from Oxoid GmbH, Wesel, Germany.

Methods: The study was approved by the ethics committee before its start (local ethical committee ref. no. 679.1.1/19). The measurements were conducted according to the principles of the World Medical Association’s Declaration of Helsinki, and all patients provided written informed consent.

To ensure a sufficiently large patient population in which results would be statistically significant, we chose a prospective study based on a power analysis. We compared patients who were followed-up with at the 1st Orthopaedic Clinic of the 1st Faculty of Medicine of Charles University in Prague and Motol University Hospital from 14 May 2020 to 10 August 2023. The cohort consisted of 155 patients (87 women and 68 men). The examined patients ranged in age from 43 to 90 years (average of 57 years).

For a comparison of the results, we followed two cohorts of patients. The first group included patients with infectious complications of hip and knee joint replacements (n = 44, TKA = 22, THA = 22); both acute and chronic infections of the replacements were included. In the second control group of patients, infection of the synthetic implant was ruled out (n = 111, TKA = 51, THA = 60). Patients who had no infection were most often examined or operated on for wear of the articulating surfaces of the replacement, joint pain, aseptic loosening of the acetabular or femoral component of the hip joint, or aseptic loosening of the femoral or tibial component of the knee joint. Patients with ongoing chronic inflammatory disease such as rheumatoid arthritis, malignancy, or an acute ongoing infection of other organs (e.g., pneumonia, urinary tract infection) were excluded from the study. A total of 85 hip replacements and 70 knee replacements were examined (Table 1).

A sample was taken preoperatively in 87 cases via puncture in the outpatient room under X-ray control and 68 times during the surgical procedure. In the case of the preoperative sample, synovial fluid was taken via puncture in 24 patients before making an incision into the joint itself. This prevented the final pH value from being influenced by bleeding from the cut. The pH measurement was performed within 2 h of sample collection and was performed by a single operator. The pH was measured at room temperature (23 °C) with a WTW inoLab 7110 pH meter and a WTW SenTixMic-D electrode (Wissenschaftlich-Technische Werkstätten GmbH, Weilheim, Germany). The pH value was measured with an accuracy of 0.01, and the electrode was disinfected with 3% hydrogen peroxide after each measurement. The electrode was kept in a KCl solution between measurements. The calibration of the pH meter was carried out monthly with three buffers (Hamilton Duracal Buffer) with pH values of 4.01, 7, and 10.01. In the case of sample collection via puncture, we took a sample of synovial fluid for culture and PCR for each patient; if we had a sufficiently large amount of fluid, it was also sent for an alpha-defensin examination. In the case of postoperative sample collection, we took at least 4 tissue samples for culture, plus one sample for PCR. Aerobic culture was performed on Columbia Agar with Sheep Blood (7%), MacConkey Agar, and Chocolate Agar with Vitox (cultivation in an environment with 5% CO_2_) (Oxoid GmbH, Wesel, Germany). Multiplication took place in Thioglycolate Broth (broth) for 24 h after inoculation and repeated on all the samples mentioned. Anaerobic culture was performed on Schaedler Anaerobe Agar (primary culture) and multiplication in the Thioglycolate Broth (Oxoid GmbH, Wesel, Germany). Prolonged culture lasted 5 days. The PCR (panbacterial) was measured using a kit from Molzym UMD-SelectNA™ CE IVD (Molzym GmbH & Co., Bremen, Germany), and isolation was performed using an Arrow (Nordiag ASA, Oslo, Norway) or Seeprep12™ (Seegene Inc., Seoul, Republic of Korea) device. We took blood tests for CRP and WBC for all patients.

Infection of the joint replacements was evaluated according to the 2018 MSIS criteria [23]. A total of 27 patients were confirmed to have an infection according to the major criteria (25× two positive cultures of the same organism, 2× sinus tract with evidence of communication to the joint), and 17 patients were confirmed to have an infection according to the minor criteria.

### Statistical Analysis

A statistical analysis was performed using the program Origin 2019 (version 9.6.0.172, OriginLab Co., Northampton, MA, USA) by means of one-way ANOVA. Probability levels of *p* = 0.05 were determined to be significant, and *p* = 0.001 was determined to be highly significant.

## 3. Results

**Figure 1:**
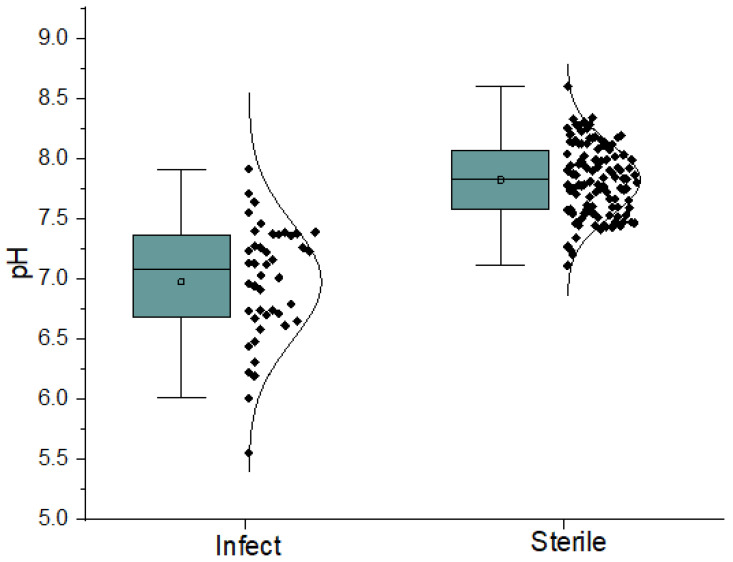
Spread of pH levels of two groups of patients through their quartiles.

The group of patients with infection (n = 44) had a lower synovial fluid pH (pH = 6.98 ± 0.48) by 0.85 pH units than the group of patients with aseptic fluid (n = 111, pH = 7.82 ± 0.29) (Figure 1). The corresponding median pH values were 7.08 for the patients with infection and 7.83 for the patients with aseptic fluid (Table 2). The difference between the group of patients with infection and the group with no infection was statistically highly significant (*p* < 0.001, F = 175.58, *p* = 3.54 × 10^−27^) (Table 3). As pH is a logarithmic value, the difference of pH 0.85 units means that the concentration changes in H^+^ ions were approximately 7 times.

The most common pathogen in the patients with infected replacements was *Staphylococcus aureus* (43.2%), followed by *Staphylococcus epidermidis* (25%), *Streptococcus agalactiae* (11.4%), and *Pseudomonas aeruginosa* (4.6%). The following pathogens were found in only one sample each: *Streptococcus mitis*, *Gardenella vaginalis*, *Streptococcus dysgalactiae*, *Anaerococcus hydrogenalis*, *Staphylococcus pettenkoferi*, *Morganella morganii*, *Streptococcus sanquinis*, *Finegoldia magna*, *Fusobacterium nucleatum*, *Enterococcus faecalis*, and *Propionebacterium avidum* (Table 4). All pathogens were confirmed through culture tests.

In four patients (9.1%), we observed a polymicrobial infection: in one patient with a knee replacement infection (*Staphylococcus aureus + Staphylococcus epidermidis*) and in three patients with hip replacement infections (*Staphylococcus epidermidis + Anaerococcus hydrogenalis*, *Finegoldia magna + Staphylococcus aureus,* and *Enterococcus faecalis + Fusobacterium nucleatum*). The average value of CRP in the patients with infection was 117.5 mg/L; in patients without replacement infection, it was a CRP of 10.3 on average mg/L. Alpha defensins were measured in 90 patients: 17 patients with infection and 73 patients with no infection. Sampling for alpha defensins was performed mainly in patients in whom we performed diagnostic punctures (n = 75). In the other 15 samples, we performed the collection preoperatively. At the 1st Orthopaedic Clinic of the 1st Faculty of Medicine of Charles University in Prague and Motol University Hospital, we introduced a quantitative method for measuring alpha defensins using high-performance liquid chromatography (HLCP). The average value of alpha defensins in patients with an infectious complication of the joint replacement was 879.9 mg/L (165–2347 mg/L). The value of alpha defensins in synovial fluid from non-infected joint replacements was 28 mg/L (0–576.5 mg/L). In 61 aseptic samples, the value of alpha defensins was zero.

Out of the total 44 infectious samples, 39 samples (88.6%) had a pH lower than that of the internal environment (7.4). Only in five cases (12.4%) was the pH higher than 7.4 (Figure 1). Out of the five samples with a pH higher than 7.4, three samples were from hip replacements, and two samples were from knee replacements. In one case, the infection was caused by *Staphylococcus aureus*, where the pH was 7.71, and this correlated with a low CRP of 19.8 mg/L. We assume that this was a mitigated chronic infection. Furthermore, in two cases, higher pH values of 7.55 and 7.46 units were observed in *Pseudomonas aeruginosa*. We attribute this to the different environments created by the bacterium. This fact was confirmed by us in a recent publication where we investigated the pH of individual bacterial cultures in laboratory conditions [24]. The *Staphylococcus aureus* decreased the pH value from 7.2 to 5.6 during 10 h, but *Pseudomonas aeruginosa* was not shown to change pH levels [24]. In another case, the pH was 7.64 in a confirmed infection of a hip replacement with *Staphylococcus coagulase negative*. This patient had a positive culture, a positive PCR, and an elevated CRP of 47 mg/L. In the last case, the pH was 7.91 in an infection of a knee joint with *Streptococcus agalactiae,* with a CRP of 46.5 mg/L.

Out of 111 samples of synovial fluid from patients without infectious complications, only 5 samples (4.5%) had a pH below 7.4 units: 3 samples were from hip replacements and 2 samples were from knee replacements. The pH values of these samples were 7.27, 7.11, 7.24, 7.20, 7.34.

When we determined the cut-off level of pH 7.4, the sensitivity level of infected replacements was 88.6%, and the specificity level of the measurement was 95.5%. The predictive value of a positive test was 88.6%, and the predictive value of a negative test was 95.5%.

## 4. Discussion

The detection and treatment of joint replacement infection have recently received much attention. A number of both serum biomarkers and markers of inflammation from the joint exudate have been investigated, some of which have reached substantial application in clinical practice [10,17,25]. However, despite considerable progress, the early diagnosis of joint replacement infection remains a challenging task, and this issue has not yet been satisfactorily resolved. Early and rapid therapeutic intervention has a crucial impact on the prognosis of treatment. The results of most of the currently used methods are available in a longer time frame, in the order of hours from sampling. One of the promising indicators of inflammation seems to be the pH level of exudate in the region of the joint replacement. However, there are not many studies in the literature that address this issue, so we decided to examine it in this study.

The decreasing tendency of pH in the area of inflammation has been known for decades. In 1937, Menkin described a decrease in the pH of the joint exudate in a region with ongoing inflammation. He reported a decrease in pH to a value of 6.5 [22]. In 1953, Ropes and Bauer published a paper in which they determined the pH value in a normal, healthy human joint to be 7.39. They compared this value with eight samples from patients with rheumatoid arthritis, where they measured the pH to be, on average, 7.22. The measurements were performed after a joint puncture outside the patient’s bodies [26]. Similar results were published by Cummings and Nordby (1966) a few years later, when they evaluated the pH of the synovial fluid of seven healthy subjects and eight patients with rheumatoid arthritis. Their results indicated the pH of healthy subjects to be, on average, 7.43 and the pH of patients with rheumatoid arthritis to be 7.22 [27]. Gobelet, in his work from 1984 [28], demonstrated a correlation between a decrease in the pH level and an elevation in the lactate level in synovial fluid. He showed the highest concentration of lactate in non-gonococcal infectious arthritis. He also observed high lactate levels in seropositive rheumatoid arthritis and crystal arthropathies. He observed moderately elevated lactate levels in seronegative arthritis, hemarthrosis, and gonococcal infections.

In his 1978 paper, Ward demonstrated a close correlation between increased leukocyte counts in differential synovial fluid in septic arthritis and a decrease in pH [29]. Differential leukocyte counts in synovial fluid are now one of the routinely used methods for diagnosing an implant infection [23,30]. Miloshev evaluated the pH of synovial fluid from 167 patient samples in 2017. The patients were divided into three groups: probands with osteoarthritis of the knee and hip (n = 101), knee and hip replacement with aseptic complication (n = 58), and knee and hip replacement with septic complication (n = 8). The results proved that the pH decreases significantly in the synovial fluid in the area of the implanted joint replacement due to increased metal levels. The pH values of the osteoarthritic joint specimens were 7.78 ± 0.38, 7.60 ± 0.31 for the revision aseptic specimens, and 7.55 ± 0.25 for the specimens with septic complication. Based on the elevated metal levels, Miloshev expected the pH to be lower, but the influence of metal ions is hindered by the buffering capacity of the human body [31]. According to Roman, the pH value of an aseptic joint with advanced osteoarthritis is slightly lower (7.35) [32] than the pH value of the synovial fluid of a healthy joint (7.43) reported today [27].

Based on the available studies, we determined the cut-off level of pH for prosthetic joint infection to be 7.44. From the measured values, we found that the pH in the aseptic complications of replacements ranges up to 8.34 units. Further studies will be needed to determine the exact level of cut-off pH in patients with prosthetic joint infection. From our data, it seems that the pH level is also influenced by the etiological agent. In our study, we recorded the pH values to be 7.55 and 7.46 in patients where *Pseudomonas aeruginosa* was identified as the cause of the prosthetic joint infection. We attribute this to the different environments created by this specific bacterium. In other cases where the pH was higher, chronic mitigated infections were diagnosed, caused by strains of *Staphylococcus aureus*, *Streptococcus agalactiase*, and coagulase negative *staphylococcus*. An unresolved query pertains to the longitudinal pH evolution in the chronic infections of joint replacements.

In the available literature, studies are beginning to appear that consider a pH measurement of the exudate as a potentially useful marker for the early diagnosis of prosthetic joint infection, as well as a potential marker of inflammation for the development of implantable biosensors. In 2021, Uthpala et al. [33] presented a design of an implantable biosensor, which they tested on bovine samples of synovial fluid. The sensor consists of a hydrogel in a polymer case, which is attached at one end with a tungsten wire, and, at the other end, there is a radiopaque tantalum ball. The evaluation of pH change is performed via X-ray imaging, i.e., a method commonly used to examine patients after joint replacement. The sensor showed very good linear reactivity to pH changes under ex vivo conditions in the range of 6.5 to 7.5 [33]. Another paper that mentions pH as a potential marker for biosensors is that by Iyengar et al. from 2021. For infection detection, they propose measuring the temperature, pH, and local biochemical changes in lactate and glucose levels [34].

In the future, our goal is to design, develop, and manufacture an implantable co-sensor that would measure pH and other values (e.g., temperature, redox potential, and defensins) in the joint replacement area at clearly defined intervals. If the pH values fall below a defined level, the information will be telemetrically transmitted, and early therapeutic intervention will be possible, increasing the chances of preserving the implanted replacement and, thus, significantly reducing the patient’s burden in the treatment of prosthetic joint infection.

## 5. Conclusions

In this study, we confirmed in a large sample of patients that a decrease in the synovial fluid pH of the hip and knee replacement area correlates with ongoing inflammation. A measurement of the pH of synovial fluid from the areas of hip and knee replacements should, in our opinion, belong to the spectra of examination methods used to diagnose replacement infection. Its advantages include evaluating the result within a few minutes of sampling the measured synovial fluid, either via joint puncture or preoperatively. Our results are consistent with those of other authors, where the pH limit of infectious samples is mostly considered to be below 7.4. Another advantage of this method is the future involvement of a sensor that detects pH values in smart replacements of knee and hip joints, which will evaluate the pH levels over time. Subsequently, their dynamics will be mainly evaluated. However, further studies will be needed to refine the application of this method in the diagnosis of prosthetic joint infection. In addition to these important results, the measurement has limitations. The measurement of pH level is not sensitive in the diagnosis of infection caused by Pseudomonas aeruginosa. Therefore, we must always take a comprehensive approach to the diagnosis of joint replacement infection and use as many diagnostic methods as possible.

## Figures and Tables

**Table 1:** Total number of infectious and non-infectious samples of knee (TKA) and hip (THA) replacements.

	Infected	Non-Infected
THA	22	60
TKA	22	51

**Table 2:** Measurement of synovial fluid samples according to pH values.

	Infected (n = 44)					Non-Infected (n = 111)			
pH	<7	7–7.2	7.21–7.4	>7.4	<7.4	7.4–7.6	7.61–7.8	7.81–8	>8
number of measurements	20	6	13	5	5	27	21	26	32

**Table 3:** Statistical analysis of two groups of patients performed by means of one-way ANOVA (*p* = 0.05 = significant, *p* = 0.001 = highly significant). Group A (44 patients)—infected; Group B (111 patients)—non-infected.

Groups	Number of Patients	Mean Value	Standard Deviation
group A	44	6.975	±0.484
group B	111	7.822	±0.295

F = 175.58, *p* = 3.54 × 10^−27^.

**Table 4:** Identified bacteria for the 44 patients in the infectious group.

Agents	Number	THA	TKA
*Staphylococcus aureus*	19 (43.2%)	4	15
*Staphylococcus epidermidis*	11 (25%)	9	2
*Streptococcus agalactiae*	5 (11.4%)	1	4
*Pseudomonas aeruginosa*	2 (4.6%)	2	0
*Anaerococcus* *hydrogenalis*	1 (2.3%)	1	0
*Streptococcus mitis*	1 (2.3%)	1	0
*Staphylococcus* *pettenkoferi*	1 (2.3%)	1	0
*Morgnella morganii*	1 (2.3%)	0	1
*Streptococcus* *sanquinis*	1 (2.3%)	0	1
*Finegoldia magna*	1 (2.3%)	1	0
*Fusobacterium* *nucleatum*	1 (2.3%)	1	0
*Propionebacterium* *avidum*	1 (2.3%)	1	0
*Enterococcus faecalis*	1 (2.3%)	1	0
*Streptococcus* *dysgalactiae*	1 (2.3%)	1	0
*Gardenerra vaginalis*	1 (2.3%)	1	0
*Polymicrobial infection*	4 (9.1%)	3	1

## Data Availability

The article’s data will be shared upon reasonable request to the corresponding author.
